# Serum fibrinogen level and fibrinogen administration in patients with traumatic brain injury: A systematic review and meta-analysis protocol

**DOI:** 10.1371/journal.pone.0310066

**Published:** 2025-07-30

**Authors:** Joanne Igoli, Jeremiah Oluwatomi Itodo Daniel, Halleluyah Oludele, Adedoyin Esther Alao, Idemudia Stephen Ogedegbe, Adewale Olaniyan, Michael Adeshola Adebayo, Damilola Matthew, Temidayo Elizabeth Oyepitan, Daniel Brabi, Olatomiwa Olukoya, Temidayo Osunronbi

**Affiliations:** 1 Deanery of Clinical Sciences, The University of Edinburgh, Edinburgh, United Kingdom; 2 Neurosurgery Department, Surgery Interest Group of Africa, Lagos, Nigeria; 3 National Hospital for Neurology and Neurosurgery, London, United Kingdom; 4 Department of Neurosurgery, Salford Royal NHS Foundation Trust, Manchester, United Kingdom; Nationwide Children's Hospital, UNITED STATES OF AMERICA

## Abstract

**Introduction:**

Traumatic Brain Injury (TBI) is a leading cause of disability and death globally. It has a significant economic burden. Coagulopathy has been identified as one of the key factors contributing to the poor outcomes observed in TBI patients, and it has been theorised that the management of coagulopathy will improve patient outcomes. Low serum fibrinogen levels denote a coagulopathic state, and the therapeutic administration of fibrinogen has been proposed to correct this state. However, there is no consensus on its efficacy in patients with TBI. Hence, this systematic review and meta-analysis seeks to ascertain the prognostic value of serum fibrinogen levels in patients with TBI and assess the effect of fibrinogen administration on these patients.

**Methods:**

Using the Preferred Reporting Items for Systematic Review and Meta-Analysis Protocols (PRISMA-P) guidelines, we will perform a comprehensive search of Scopus, Medline, Embase and Cochrane Library to retrieve all original articles that investigate the prognostic value of fibrinogen levels and/or the effect of fibrinogen administration in TBI patients. Primary outcomes include functional outcome and mortality assessments such as the Glasgow Outcome Score and modified Rankin Score. Secondary outcomes include progressive intracranial haemorrhage/contusion and need for surgical intervention. Data collected will encompass participant demographics, measured fibrinogen levels, dose of fibrinogen administered and specified outcome measures.

**Conclusion:**

Findings from this study, specifically the evidence if fibrinogen level has prognostic value and if fibrinogen administration improves patient outcomes, will help inform future TBI management. It will also enhance shared decision-making between healthcare professionals and patients if fibrinogen has a prognostic value, as this value could be used to communicate more effectively the expected prognosis post-TBI. Thus, TBI patient outcomes can be optimised accordingly.

**PROSPERO Registration Number:**

CRD42024556497. Available from: https://www.crd.york.ac.uk/prospero/display_record.php?ID=CRD42024556497

## Introduction

Traumatic Brain Injury (TBI) is a leading cause of disability and death, with an incidence of approximately 69 million people per year globally [1] and a prevalence of approximately 75 million [1,2]. The estimated global economic cost of TBI is $400 billion [3]. Approximately half of TBI patients seen in hospitals fail to regain pre-TBI baseline health six months after the injury [2]. Sequelae of TBI range from transient loss of consciousness and confusion to seizures, persistent cognitive decline, coma, and death [4–6].

Therefore, it is important to accurately prognosticate TBI patients to better tailor treatment strategies. Current methods used to prognosticate TBI patients include Glasgow Coma Score (GCS), pupillary response and CT scan findings [7,8]. Given acceptable correlation with patient outcomes, these have been adopted in clinical practice for treatment decisions, for example withdrawing treatment in TBI patients with bilaterally fixed and dilated pupils [9]. However, these factors on their own do not fully explain the variation in outcomes seen in TBI patients. Hence composite prediction models such as the International Mission for Prognosis and Analysis of Clinical Trials in Traumatic Brain Injury (IMPACT) have been proposed [7,10]. Discrepancies between the outcomes seen in clinical practice and predictions of these models exist [11]. Therefore prognostic biomarkers have been proposed as important adjuncts to these that can improve TBI prognostication [11].

Coagulopathy in TBI influences the development of intracranial haemorrhage (ICH), leading to poor patient outcomes, including a higher incidence of organ failure, poor neurological function recovery, significant disability, and death [12–14]. Compared to other clotting factors, fibrinogen level is routinely measured in clinical practice and is an inexpensive marker of coagulopathy [15]. Fibrinogen plays a vital role in the clotting cascade of stabilising blood clots. A low fibrinogen level is a marker of coagulopathy seen early in TBI patients with fibrinogen consumption being a key mechanistic step in TBI related coagulopathy [16]. Serum fibrinogen level has been shown to decrease significantly hours after trauma and reaches severely low levels in uncontrolled post-traumatic bleed [17]. This uncontrolled post-traumatic bleed is a potential mechanism through which low fibrinogen levels lead to poor outcomes of loss of independence due to severe disability, vegetative state, and death [14,18,19].

It has been postulated that low fibrinogen levels can be used to prognosticate patients with TBI, and the administration of fibrinogen in the correction of hypofibrinogenemia could improve outcomes in these patients [20]. However, there is conflicting evidence in the literature regarding the prognostic value of fibrinogen level or benefit of fibrinogen administration in TBI patients.

Consequently, this planned systematic review aims to analyse the existing evidence and evaluate the relationship between baseline fibrinogen levels and outcomes in TBI patients, with a view to improving risk-benefit considerations and shared-decision making in the management of these patients. Secondly, the authors aim to evaluate the evidence on the benefits, if any, of fibrinogen administration in patients with TBI. This, if demonstrated, would transform and broaden treatment options in this group of patients.

## Materials and methods

This study protocol was designed following the Preferred Reporting Items for Systematic Review and Meta-Analysis Protocols (PRISMA-P) guidelines (S1 file).

### Research questions

Question 1: Is there a prognostic value of fibrinogen level in patients with traumatic brain injury? (Table 1).

**Table 1:** PICO table – Research question 1.

*Population*:	Patients with traumatic brain injury (TBI).
***Intervention*:**	Serum fibrinogen level.
***Control*:**	N/A.
***Outcomes*:**	Glasgow outcome score; need for surgical intervention; mortality; functional outcomes; progressive intracranial haemorrhage/contusion; other.

Question 2: What is the effect of fibrinogen administration on outcomes in patients with traumatic brain injury? (Table 2).

**Table 2:** PICO table – Research question 2.

*Population*:	Patients with traumatic brain injury (TBI).
***Intervention*:**	Fibrinogen administered.
***Control*:**	Fibrinogen not administered.
***Outcomes*:**	Glasgow outcome score; need for surgical intervention; mortality; functional outcomes; progressive intracranial haemorrhage/contusion; other.

### Data collection

#### Literature search strategy.

We searched the abstracts and titles of articles in Medline, SCOPUS, Cochrane, and EMBASE databases from inception to April 18, 2025 using the following search strategy:

#1 head OR craniu* OR crania* OR cerebra* OR cerebru* OR brain* OR forebrain* OR skull* OR hemispher* OR intracran* OR intracerebral
#2 injur* OR trauma* OR damag* OR wound* OR fracture* OR contusion* OR haematoma OR hematoma OR haemorrhage OR hemorrhage
#3 Factor I OR Fibrinogen OR Fibrin OR Fibrinogen concentrate OR Hypofibrinogenemia OR Hypofibrinogenaemia OR Fibrin degradation product OR Coagulopathy OR Clot* OR Haematolog* OR Hematolog* OR Cryoprecipitate OR Riastap OR Fresh Frozen Plasma
#4 #1 AND #2 AND #3

The initial results of the search strategy are shown in Table 3.

**Table 3:** Search strategy table.

Database	Count
Scopus	40,451
Medline	8,652
Embase	15,828
Cochrane	2,626

### Eligibility criteria

#### Inclusion criteria.

We will include full-text original articles that investigate the prognostic value of serum fibrinogen level and/or investigate the effects of fibrinogen administration on outcomes in TBI patients. These articles will include randomised controlled trials (RCTS), observational studies such as cohort studies and case-control studies. We would also include grey literature specifically relevant pre-print studies, theses and dissertations.

#### Exclusion criteria.

Review articles, expert opinions, editorials, letters, conference abstracts, case reports, case series, non-English language papers, paediatric-focussed studies and non-human studies will be excluded. Studies in which fibrinogen was used solely as a marker of disease severity and/or solely for diagnostic reasons will also be excluded.

### Data extraction

Zotero (Corporation for Digital Scholarship, Virginia, USA) will be used to exclude literature search duplicates. Using Rayyan (Rayyan, Cambridge, Massachusetts, USA), titles/abstracts will be independently screened by two reviewers for inclusion. Eligible full-text articles will be screened further, and subsequent data extracted using the same data extraction proforma on Google Sheets (Google LLC, Mountain View, California, USA). A third reviewer will adjudicate any conflicts in title/abstract screening, full-text screening, and data extraction. The process will be illustrated using a Preferred Reporting Items for Systematic Reviews and Meta-Analyses (PRISMA) [21] flow diagram. Additionally, a PRISMA 2020 checklist and PRISMA 2020 extension for abstract checklist will be provided with publication of the completed study.

**Data to be extracted:**

- Title
- Author
- Publication year
- Study design
- Country of study - based on where the study took place. The first author’s country will be used when the study location is not specified
- Cause of TBI, if included, such as blunt trauma, penetrating trauma, other
- Neurosurgical procedures carried out such as decompressive craniectomy or craniotomy for extradural clot evacuation
- Exclusion criteria
- Number of patients
- Age (mean ± SD/median (IQR))
- Sex (male/female proportion)
- Day serum fibrinogen blood sample was obtained
- Fibrinogen level (mean ± SD/median (IQR))
- Cut off value defining low Fibrinogen level
- Level of serum fibrinogen that triggered fibrinogen administration (if serum fibrinogen was checked pre-fibrinogen administration)
- Day fibrinogen was administered - preoperative or postoperative. If postoperative, the day(s) fibrinogen was administered will be extracted
- Dose(s) of fibrinogen administered
- Number of times fibrinogen was administered and the triggers for subsequent administration
- Outcome measure - the nature of the outcome measure to be identified is uncertain. Therefore, there will be no prior criteria. It is anticipated that outcomes such as the following will be identified and used as inclusion criteria:
  - Glasgow Outcome Score (GOS)/Glasgow Outcome Score Extended (GOSE)
  - Modified Rankin Score
  - Mortality
  - Quality of life
  - Progressive intracranial haemorrhage/contusion
  - Other

For each outcome measure, the following will be extracted:
  - Length of stay (LOS)
  - Number of patients with event/number without event
  - Length of follow-up
  - Number of patients lost to follow-up
  - Low serum fibrinogen threshold
  - The method used for threshold selection
  - Area under the curve (AUC)
  - Sensitivity
  - Specificity
  - Event ratio with 95% confidence interval (CI)
  - Conclusion (on the association between fibrinogen level and outcome measure; on association between fibrinogen administration and outcome measure)

### Data analysis

The direction of effect for the association between serum fibrinogen level or fibrinogen administration and patient outcomes will be categorised as significantly positive, significantly negative, or not statistically significant from articles where the event ratio is reported. Where the area under the curve (AUC) is reported, the effect direction will be grouped as failed (AUC = 0.5 to 0.6), poor (AUC = 0.6 to 0.7), acceptable (AUC = 0.7 to 0.8), good (AUC = 0.8 to 0.9) and excellent (AUC > 0.9) [22]. For event ratio, adjusted estimates would be preferred to unadjusted estimates if both are provided. Unadjusted estimates will be used however, if no adjusted estimates are available. Analysis will be run with unadjusted estimates included and sensitivity analysis will be conducted with adjusted estimates only. Dose and response analyses will be done [23], where possible, to evaluate the most effective dose of fibrinogen administration. Side effect frequency and severity will be reported.

Where possible, if ≥5 appropriate articles, meta-analysis will be performed using Review Manager (RevMan) (Copenhagen: The Nordic Cochrane Centre, The Cochrane Collaboration) whilst meta-regression will be conducted using OpenMeta[Analyst] software (Centre for Evidence-Based Medicine, Brown University, Rhode Island, USA). Each outcome measure will have a pooled extracted odds ratio (OR)/hazard ratio (HR) with the corresponding 95% confidence interval (CI). Where possible, the mean difference of fibrinogen level will be calculated as an effect size to aid comparison between patients with or without the noted explored events or outcome measures. The generic inverse-variance method with random-effects model will be used to assess the association between fibrinogen level/fibrinogen administration and the dichotomous outcome measure. Figures of summary receiver operating characteristic (SROC) and Forest-plots will be generated to illustrate the dispersion of effects across the included studies. Studies with missing fibrinogen levels or inconsistent outcome reporting will be included in the analysis and a separate analysis will be conducted with these studies removed. For studies on fibrinogen dosing, if there is too wide a range for dosing used, study subgroup analysis will be performed grouping studies with similar dosing together.

The Cochrane Q-statistic and I^2^ statistic tests will be used to assess heterogeneity among included studies whilst the 95% Prediction Interval (95% PI) will be used to assess dispersion of effects across included studies. The 95% PI will be calculated using CMA Prediction Intervals software (Biostat, Incorporated, New Jersey, USA). The PI will be calculated using these four parameters: (1) OR, (2) upper bound of 95% CI, (3) Tau^2^, and (4) number of included studies. The heterogeneity will be explored for potential sources of heterogeneity by subgroup analyses and meta-regressions. For subgroup analysis, studies will be stratified by injury severity (mild, moderate and severe TBI) and the world bank country economic groups of High-Income-Countries (HICs) and Low-Middle-Income-Countries (LMICs) which has been shown to affect TBI patient outcomes [24]. Meta-regression analysis will also be stratified using the following: mean/median age, patient gender-ratios (Male/Female) and injury severity (mild, moderate and severe TBI).

Where possible, a Trial Sequential Analyses (TSA) will be conducted using the TSA software (Version 0.9.5.10 Beta, Copenhagen University Hospital, Denmark) to quantify the statistical reliability of data in the cumulative meta-analyses. Similarly, outlier analysis will be performed using the Jamovi software (Version 2.6, Syndey, Australia). Sensitivity analyses will be carried out for studies that explicitly state that their study participants had isolated head injuries, included both paediatric and adult patients, and included patients already on anticoagulants. Sensitivity analyses will be further conducted by repeating the primary analysis after excluding studies with a high risk of bias. The risk of bias (RoB) in the eligible full-text articles will be assessed by two independent reviewers, with a third reviewer adjudicating any discrepancies.

The RoB in studies investigating the prognostic utility of serum fibrinogen level will be assessed using the Quality in prognosis studies (QUIPs) tool [25]. The categories of high, moderate, and low risk of bias will used based on the criteria developed by Grooten et al [25]: green (low risk of bias) for studies where all six QUIPs domains are assessed as low risk of bias (RoB) or not more than one domain is assessed as moderate risk of bias; red (high RoB) for studies where ≥ 1 is assessed as high RoB, or ≥ 3 domains are assessed as moderate RoB; yellow (moderate RoB) for all other articles in between. The same criteria will be used to assess the risk of bias rating for individual domains.

For the studies investigating the efficacy of fibrinogen administration, the Cochrane Risk of Bias tool will be used for Randomised control trials (RCTs) [26], and the ROBINS-I tool for non-randomised studies to determine the RoB [27]. The RoB of RCTs will be rated as high, low, or unclear. A green circle with a ‘+’ symbol within will be used for low RoB, a red circle with a ‘-’ symbol within for high RoB, and a yellow circle with a ‘?’ symbol within for unclear RoB. For the ROBINS-I tool the categories used will be low risk, moderate risk, serious risk, and critical risk of bias [27]. Publication bias will be assessed through funnel plots and Egger’s regression test, contingent on having an adequate number of studies. The GRADE framework will be used to assess the certainty of the evidence of the synthesis findings for each outcome measure in cases of fibrinogen administration and prognostication [28,29]. Two independent reviewers will conduct the GRADE assessments, with a third reviewer adjudicating any discrepancies.

If meta-analysis is not possible, the Synthesis without meta-analysis (SWiM) in systematic review reporting guidelines will be used to report narrative and qualitative data [30].

### Study limitations

Due to the nature of this systematic review’s exclusion criteria, potentially valuable information in these excluded studies may be missed. Potentially valuable information from the data in studies that fail to report the events ratio or area under curve (AUC) whilst assessing the prognostic value of fibrinogen will also be missed as the data in these studies will not be included in our data analysis.

## Conclusions

Given the prevalence of TBI and the worldwide economic burden, optimising the care of TBI patients is pertinent. If fibrinogen has a prognostic value, it would help healthcare teams practise realistic medicine in adjusting accordingly the goals of care delivered to these patients based on their prognosis.

If administering fibrinogen does indeed improve patient outcomes, then this would be an important treatment option for healthcare teams to consider incorporating for TBI patients, especially if fibrinogen administration is protective against haemorrhagic transformation of contusions.

Therefore, if we find that serum fibrinogen level has prognostic value and that administering fibrinogen improves outcome, this could be important in shared-decision making and in ensuring that patients have improved outcomes.

## Supporting information

S1 File Fibrinogen in TBI protocol PRISMA-P-SystRev-checklist. S1 showing checklist according to Preferred Reporting Items for Systematic Review and Meta-Analysis Protocols (PRISMA-P) guidelines which guided the design of this protocol.
(PDF)

S2 File Fibrinogen in TBI systematic review PLOSOne checklist. S2 showing PLOSOne  Human Participants Research Checklist.
(DOCX)
